# Teaching Immunology as a Liberal Art

**DOI:** 10.3389/fimmu.2020.01462

**Published:** 2020-07-14

**Authors:** Devavani Chatterjea

**Affiliations:** Biology Department, Macalester College, Saint Paul, MN, United States

**Keywords:** immunology education, undergraduate, liberal arts, storytelling, metaphor

## Abstract

A complex, rapidly evolving biomedical field that is of critical relevance to human health and well-being, immunology provides important and substantive opportunities to practice and teach the central tenets of a liberal arts curriculum.

## Gallery Day

It's one of those “end of semester” days in December—I am looking forward to wrapping up the term, the familiar mix of exhaustion and anticipation in my bones. The junior and senior biology majors in my immunology survey course at an undergraduate liberal arts college in the Midwest are setting up their immunology-themed art presentations. A pile of “plushies”—giant stuffed fabric white blood cells decorated with their known surface markers invites tactile exploration, and an impromptu game of toss. An immune cell synapse wired with LEDs lights up in series as “activation” switches are flicked on. Students flock to the edible displays. A towering croquembouche “lymph node” of choux pastries invites them to pull out individual ones to taste—flavored with different fillings, the pastries represent the different cells in a lymph node. As the puffs get eaten, the spun sugar matrix of the tower loses shape, much as a lymph node matrix would without resident cells. The hematopoiesis cookie table is a hit. The student who set it up explains how a basic set of ingredients is flexibly transformed into different kinds of cookies—at which points commitments to certain final products occur and when and how steps become irreversible; class-mates sample some of the finished products and take turns building cookies of different “lineages” with nuts, fruit, chocolate chips, bits of candy sparking a spontaneous discussion about food allergies, routes of exposure and safe handling practices. A student clears their throat and the hum of chatter subsides. A self-described “non-artist,” they have chosen instead to deliver a “testimony to Congress” to advocate for robust funding for immunological research inspired by the advocacy of members of the American Association of Immunologists (1). As stand-in lawmakers, we listen attentively to the evidence-based arguments for the importance of basic immunology research for a healthy society. There are tough but respectful questions on animal research ethics, a plausible timeline for a universal flu vaccine and the structural inequities of access to cutting edge cancer therapies such as CAR-T cells. After the Q&A, students read each other's artist's statements, take turns trying to sit on the fold-out monocyte chair without falling, and play with the stick and string co-stimulation maze which can only be solved with 3 manipulations in the correct sequence!

## The Perfect Liberal Art

Over 20 years ago, I was an undergraduate in an immunology class, irresistibly drawn to the discipline despite the confounding maze of nomenclatures, the alphabet soup of transgenic TCR names and the flood of cell types and molecules that went over my head. Through graduate and post-doctoral work, my love for the field endured and I realized that I wanted an undergraduate liberal arts curriculum to be the canvas for my immunology teaching and research. I don't think that, at the time, I could have foreseen a class period quite like the one I just described: students making the material their own in inventive and surprising ways, going confidently into the heart of foundational and cutting-edge concepts and using their intellectual and practical engagement with the material to connect their study of immunology with their lives. Teaching and learning immunology as a liberal art together with my students has been transformative for all of us.

Macalester College is an urban small undergraduate liberal arts college with ~2,000 students−30% students of color, 14% international, and 16% first-generation. Biology is one of the top 5 majors. I teach an immunology survey course with laboratory for undergraduates who have taken cell biology and genetics. Though the human immune system is the primary focus of the course, we study amoeba, social insects, bacteria, plants, and jawless fish to better understand the evolution of protective responses. Students write multiple-draft review papers with graphical abstracts, volunteer with the Immune Deficiency Foundation, present art/performance works and write weekly reflective essays connecting their immunology learning to other parts of their academic or personal lives. My immunological methods course is embedded in my research program investigating the connections between environmental toxins, allergic responses, and chronic pain; students participate in scientific conversations and critique scaffolded by preparatory writing assignments, map meta-arguments from sub-fields of published literature, cooperatively design, and execute experiments, and write a collaborative scientific paper. I use my upper-level seminar courses—Neuroimmunology and Cancer Immunology to teach more advanced students about public communication of science. In our college's First Year Course program I offer semester-long immunology-themed courses: *AIDS/Influenza/Malaria - ancient pathogens in a brave new world* explores the persistence and re-emergence of infections and inflammatory diseases in vulnerable populations around the world and *Bodies on Fire* centers on the global pandemic of inflammatory diseases. These courses do not have pre-requisites and are structured around connecting patient/physician memoirs, popular science books, and science journalism with the scientists and scientific discoveries they describe and typically ask students to explore these connections through writing, movement, and art.

Historian William Cronon describes the essence of liberal education as “gaining the power and the wisdom, the generosity, and the freedom to connect”—through the acts of listening, reading, writing, talking, solving puzzles, seeking complex truths, seeing other perspectives, working in a community and being willing to both lead and follow in honest and imaginative ways (2). Structurally, a liberal arts education connects the natural and physical sciences, humanities, social sciences, quantitative thinking, and artistic inquiry. Even as they engage deeply with methods and analyses in particular areas of study, students learn to appreciate different ways of making meaning of our world with tools from different disciplines. They learn to recognize and interrogate the societal structures and deep assumptions that drive the ways in which such bodies of knowledge are constructed within and across academic disciplines.

Immunology is a perfect fit for a liberal arts education. While traditional practices such as variolation and uses of immunomodulatory foods and botanical medicines have existed for thousands of years in societies around the globe, the constructs of cellular and circulating immune mechanisms have been articulated in the context of academic biomedicine only as recently as the late 1800s. And within these 200 years, paradigms have been swiftly proposed, critiqued, modified and transformed into an ever more complex and nuanced understanding of immunity (3). Concepts of preservation of self over “non-self” have morphed into understandings of danger, disruption, repair, and memory embedded deep within cell lineages, epigenetic imprints and tissue architectures. Mechanisms once described more bluntly as “killing pathogens” are now understood as highly regulated, selective, tunable responses to commensal and non-commensal microbes that constitute the multi-species ecosystems of multicellular hosts. While the immune system gives us critical protection for survival, virtually every global health concern from emerging infections, allergies and asthma, autoimmunity, chronic pain, and other psychiatric, cardiovascular, and metabolic imbalances are all fueled by these same mechanisms of inflammation, shifted by context to become harmful and pathological. Author Chimamanda Ngozi Adichie, in her TED Talk “*The Danger of a Single Story*,” warns that assuming a single story about a people leads to dangerous misconceptions, and learning to listen for the many different stories is essential for cross-cultural understanding (4). Immune responses, with their double-edged nature, provide a natural set of case studies in the importance of “many stories.” Immune responses demand careful contextual analyses, and to study them closely is to learn to grapple with complexity and uncertainty—an essential skill in today's rapidly changing, connected yet divergent world.

## Toolkits for Life, Work, and Storytelling

Another advantage of studying immunology is its immediate personal and social relevance. Students only have to look at their own bodies, experiences of well-being and illnesses, and their environments for applications of what they learn. For many students, one immunology-related class might be their only sustained experience with the discipline, but the lessons they draw from it have the potential to remain relevant and useful in their lives. As a powerful example of this, I have observed my Neuroimmunology students particularly resonate with learning about the role inflammation plays in mental health. Students on college campuses are experiencing anxiety and depression at unprecedented levels, and managing neurological diagnoses while removed from their families and support systems (5). Understanding the roles of pathological inflammation intertwined with these mental health conditions, exploring the connections of stress, diet, and rest to these neuro-inflammatory pathways are empowering for students; appreciating the “bodily” basis of psychological challenges appears to make them seem more tractable. While these lessons do not take the place of the counseling and/or psychiatric support they or their peers need and receive, I have observed that students do find this scientific parsing of the mind-body connection to be of practical use.

Many immunology students are drawn to careers in biomedical research and its applications in the practices of medicine and/or public health. Immunological research—discovery, translational, academic, clinical, industrial—and its applications in drug development, medical technologies, and public health interventions are at once scientific and social endeavors. Countering anti-vaccine movements, crafting community, and government public health responses to disease outbreaks, regulating environmental toxicants in food, water, and air all contain important immunological arguments at their core. Being able to understand and speak the language of immunology and tell its stories to specialist as well as general audiences so they can be truly heard is an important skill for students to practice. Iteratively learning to read the often dense and technical immunological literature and synthesizing and communicating these findings in their own written and spoken words is both preparation for future work in biomedical fields and a core tenet of a liberal arts education—the importance of listening, reading, speaking, arguing, and writing. These skills are not unique to the study of immunology, but immunology offers undergraduates and their professors in a liberal arts context a rich and pragmatic field within the biomedical sciences in which to practice them. Students in my courses and research laboratory write literature reviews, give talks and present posters on their research at conferences, and collaborate with me on writing papers and grant proposals for scientific audiences. However, they also write white papers and reflective essays connecting their learning in immunology to other disciplines, prepare educational materials for community organizations, teach secondary school students and mentor younger peers and, in doing so, practice translating the technical jargon of scientific communication into information that their audiences need and can use.

A spacious liberal arts education makes room for multi-disciplinary training, provides opportunities for immersive learning and community engagement and asks students to connect their learning to the world in *different* ways, giving them opportunities to make this complicated and compelling field their own. The perceived “difficulty” of immunology can be deconstructed in this more permissive, integrative environment to allow creative strategies for making meaning and finding purposeful engagement with the subject.

## Maps and Metaphors for a World in Crisis

Immune systems are synergistic wholes of interconnected parts continuously stirred up by new discoveries that complicate existing knowledge and demand new ideas and interpretations; this has been so since Paul Ehrlich sketched his intricate visions of cells shedding neutralizing anti-toxins and butted heads with Ilya Metchnikoff's cheeky but utterly prescient observation that immunity might just look like hungry amoeba out to forage (6). In the last two decades, our view of the immune system has been transformed by newly discovered innate cell subsets, the regulation of immunity by microbial and viral symbionts, the control of immune responses by metabolic switches, and the realization that all cells, not just the ones that we recognize as immune cells, participate in and regulate immune responses of multicellular organisms. This framework of synergistic interactions and multi-factorial outcomes can provide our students with maps and metaphors useful beyond immunology, for broader understandings of complex social and planetary processes.

The precarious balance of protective vs. harmful immune responses is a mirror of the collateral costs of inequities, state-sanctioned violence, and xenophobia in our societies. Chronic inflammation and accompanying adverse health outcomes are materially correlated with lower socio-economic status, lack of access to nutritious foods, stressful living conditions and unstable access to healthcare (7). That any immune response takes a toll on the living tissue it is trying to protect from real or imagined threats parallels the effect that xenophobic, reactive intolerance, and unregulated violence can have on a community or society at large. Just as our own cells and those of our commensal symbionts maintain a collaborative understanding that we disrupt at our peril, our local and global communities are sensitive to the behavior of individuals and cooperation between the diverse populations who live in them. Tolerance, balance, homeostasis, repair are technical terms with specific immunological meanings that are just as relevant to our social fabric as they are to our understanding of healthy and disease states of our bodies. And likewise, jingoistic militarized language about the immune system vanquishing pathogens can echo intolerant social rhetoric. The nuance and care required to understand and modulate immune responses and their outcomes serve as object lessons in how we speak and act as individual and collectives in social and political arenas.

An immunological framework can also be applied to the relationship of humans with our planet as a whole. Human-induced climate change has driven our planet and its inhabitants to a perilous state of imbalance, with rapid rise in temperature and sea levels, catastrophic weather events, heat stress, food shortages, displacement of peoples, biodiversity loss, emerging pathogens (such as SARS-Cov2), and exacerbation of poverty and conflict, all of which create negative health outcomes for those who are most vulnerable and have the least access to resources. The United Nations Intergovernmental Panel on Climate Change (8) advocates for immediate, massive, and collective action to mitigate this crisis if we are to survive. Our students are joining their climate activist peers—Greta Thunberg, Isra Hirsi, Xiye Bastida, and others in climate strikes and actions to emphasize the urgency of the situation. The literal health effects of climate change are, and will be marked by inflammatory processes in our individual bodies, and sharp increases in global disease burdens; it is as if the entire planet is in a state of chronic inflammatory distress. Everything is connected and what we do individually, and collectively, to our bodies and to our world comes back to us. Teaching about our immune systems in integrative, socially relevant ways can help our students make meaningful connections between the content of their learning and the larger global context in which they live.

## Beyond Information

In her book *Teaching to Transgress* (9), feminist author and educator bell hooks says:

*To educate as the practice of freedom is a way of teaching that anyone can learn. That learning process comes easiest to those of us who teach who also believe that there is an aspect of our vocation … is not merely to share information but to share in the intellectual and spiritual growth of our students*.

In information-dense, rapidly evolving fields of study, it is natural to feel overwhelmed by the responsibility to share information as accurately and comprehensively as possible before our limited time with any one group of students comes to a close. I am grateful that immunology—the beautiful, maddening, messy field that it is—keeps me humble and honest about the work I really want to do with my students and the way in which I want to do it. It resolutely refuses to be told as a “single story” and any arcane details memorized for exams are known to have modest shelf lives in any case. So with each passing year, I am challenged to re-imagine how I can best help my students be as prepared as possible to hear and understand all of the immunological stories that have not been written yet—to be able to know the workings of their future bodies and minds a little better, to understand and appreciate why a pandemic coronavirus can ravage one body it infects and leave another unscathed, to be able to use these stories to build healthy lives and communities, and make new discoveries.

In her more recent book, *Teaching Community: A Pedagogy of Hope* (10), bell hooks says: “It is imperative that we (teachers) maintain hope even when the harshness of reality suggests otherwise.” I take these words to heart. Much of western biomedical science has been built around concepts of illness rather than wellness and I wonder whether it is simply too overwhelming to keep coming back to narratives and mechanisms of morbidity, dysfunction, and imbalance. Here again, the spaciousness of a liberal arts framework allows both instructors and students to be more open to leavening the difficult topics with moments of beauty and fun. Psychologist Alison Gopnik has demonstrated that children who “pretend play,” in elaborate, unreal scenarios with the aid of language, props and gestures, are able to respond correctly to counterfactual questions about a novel real-world causal relationship (11). While the evolutionary imperative for play may well be to develop robust cognitive functions, children play because it is a lot of fun. The paradox of play is that in order to be able to reach a variety of practical benefits in the long run, one must be somewhat less concerned with immediate accomplishments of goals in the short run. Eating a cardamom and orange cream-filled choux bun pulled out of a patisserie “lymph node” might not immediately seem central to learning about immune systems but it is delicious and it distills the joy of learning and sharing in a way that sticks in our brains and hearts—both my students' and mine. A liberal arts education with its emphasis on connective and integrative inquiry aims to be transformative, to crack the world open a little bit wider for every student with every course of study, every class, every discipline. But it is not only the student who is transformed, it is also the teacher. Teaching immunology as a liberal art has made me a more curious, capable and happier immunologist than I had known I could be.

## Data Availability Statement

The original contributions presented in the study are included in the article, further inquiries can be directed to the corresponding author.

## Author Contributions

The author confirms being the sole contributor of this work and has approved it for publication.

## Conflict of Interest

The author declares that the research was conducted in the absence of any commercial or financial relationships that could be construed as a potential conflict of interest.
